# Of Tax, Crime, and Happiness. An Interdisciplinary Research Review on Social Inequality

**DOI:** 10.1007/s42520-022-00413-2

**Published:** 2022-03-02

**Authors:** Jenny Pleinen

**Affiliations:** Lehrstuhl für Neuere und Neueste Geschichte, Universität Augsburg, Augsburg, Germany

**Keywords:** Social inequality, Redistribution, Ungovernability, Opportunity hoarding, Soziale Ungleichheit, Umverteilungspolitik, Unregierbarkeit, Chancenmonopolisierung

## Abstract

This article reviews research literature on inequality from economics, sociology and political science from a historiographic perspective. The literature covered here mainly focusses on the development of Western societies between 1970 and 2010 and is organised in terms of research concepts such as tax competition, educational attainment gaps, educational assortative mating, opportunity hoarding, happiness inequality, politics of resentment, welfare chauvinism and nature versus nurture.

## Abstract (de)

Der vorliegende Artikel bespricht Werke der wirtschaftswissenschaftlichen, soziologischen und politikwissenschaftlichen Ungleichheitsforschung aus der Perspektive einer problemorientierten Zeitgeschichte anhand relevanter Forschungskonzepte. Berücksichtigt werden vor allem Aufsätze und Monografien zur Entwicklung westlicher Industriegesellschaften zwischen 1970 und 2010.

## Introduction

“Inequality is a bit like cinnamon—you definitely want to have a little of it to spice life up a bit, but too much of it can be very dangerous. And make no mistake, we are at cinnamon-dangerous levels right now”.^1^ This simile for the gap between rich and poor in America—accompanied by YouTube clips, which had gone viral, of people spewing cinnamon at the camera while doing the ‘cinnamon challenge’—featured on HBO’s satirical show “Last Week Tonight with John Oliver” in July 2014. There can be little doubt that since the global financial crisis of 2008, criticism of the detrimental effects of capitalism has become a staple of American popular culture, especially when directed towards a millennial audience. The vehemence with which perceptions of social inequality as a high-priority political problem have moved from the left into the political middle only highlights the lack of effective government policies to address this issue. The political relevance of social inequality has gained further momentum due to the spread of COVID-19. Around the world, the pandemic has highlighted dividing lines within modern societies, many of which are the result of or at least exacerbated by an unequal distribution of income and wealth: Gaps between those who have sufficient access to the internet as crucial meta-resource for remote learning and communication and those who do not as well as between those who can work from home and those who have little choice but to take increased health risks in order to earn a living, to name just two examples that received much less attention before 2020.

Against the backdrop of these developments and the resulting political relevance, multi-discipline research into social inequality has spiked and received a high level of public attention. The most prominent example might be Thomas Piketty’s “Capital in the Twenty-First Century” which—when published in English in 2014—turned a regular academic economist into *the* global “inequality guru”. Piketty’s work offered a plausible, well-documented and readable explanation of the explosive increase in social inequality we have witnessed since the 1970s and suggests how this could be countered through a global capital gains tax.^2^ Along with British economist Tony Atkinson, he also facilitated and encouraged further research by making his data freely available on “The World Inequality Database” (wid.world). As well as attracting much praise and publicity, Piketty’s work has also become the target for some sharp methodological criticism.^3^ At least part of the criticism is directed at the fact that Piketty tries to bridge history and economics—two disciplines with widely differing conventions. Historians take exception to how economists use historical data without carefully reconstructing the circumstances of data production in favour of seemingly coherent time series. The following article provides a review of research literature on inequality which crosses the borders of academic disciplines. The literature covered here mainly focusses on the development of Western societies between 1970 and 2010 and is organised in terms of research concepts. Despite methodological and cultural differences between history on the one hand and economics, sociology and political science on the other, maintaining a dialogue and paying more attention to each other’s research would enhance our knowledge of complex topics like social inequality.

## Toothless Nation-States? The Limits and Inefficiencies of Policies of Redistribution

Since the 1950s, the scope of government intervention has expanded significantly in all Western democracies. Welfare states, in particular, found new policy targets, covered additional life risks—albeit at different rates of replacement—and claimed an increasing proportion of growing public finances.^4^ This period of state expansion is usually seen as a golden age, with little regard for its brevity or disparate developments.^5^ During the 1970s, increasing societal complexity (or the perception of it) and mass unemployment put a strain on this new breadth and depth in state intervention, giving momentum to crisis discourses from both the political right and left.^6^ By the mid-1980s, scholars and political commentators doubted the state’s capacity to redistribute income and wealth effectively under the pressures of globalisation.^7^ Historian Walter Scheidel even argues that in the long run, only wars, revolutions, failing states and medical catastrophes like the bubonic plague have interrupted the tendency towards ever-growing inequality due to their shock effect.^8^ In his book “Capital and Ideology”, Piketty offers a less deterministic interpretation of post-1970s developments that is still a narrative of interrupted progress.^9^ He identifies three causes for the retreat of social democracy in Western countries: the abandonment of public ownership in the economy, the failure to consistently tax wealth with progressive rates and the failure to provide egalitarian educational systems. Poornima Paidipaty and Pedro Pinto have argued that Piketty’s argument is strong in description but does not identify factors that actually explain the failure of progressive politics or the public support for market-oriented policies.^10^ The following section explores two concepts that have been put forward as reasons for the decreasing efficacy of government redistribution: first, tax competition and other factors that discouraged states from taxing high incomes; and second, unintended changes in behaviour, like opportunity hoarding in public education.

### Tax Competition and Tax Havens

Since the 1990s, there has been an ongoing debate about whether states are in a global competition for tax revenue, which forces them to levy lower and lower taxes (“race to the bottom”).^11^ Several factors have combined to make the issue of tax competition relevant again. The deregulation of financial flows, free-floating exchange rates and relatively low tariffs allowed it to extract value from national tax regimes while taxes were high enough to make tax evasion financially attractive and worth the low risk of detection.^12^

The debate on tax competition in part describes the phenomenon in almost universal terms, as if it is a strategy relevant to all income groups and exerts pressure on all kinds of tax revenues. The empirical literature demonstrates, however, that when average or below-average income earners cross borders, by far the main reason is for higher wages, and not to avoid or lower their tax burden.^13^ The situation is different for financial services and capital income, which strongly react to taxation rates. The relative fluidity of capital gives small countries the opportunity to establish themselves as “tax havens”, especially for anonymous shell companies from the major advanced economies.^14^ Relative to their small budgets, even a low capital or corporation tax rate generates significant revenue and makes this strategy worthwhile for Switzerland, Luxembourg, the Channel Islands and several Caribbean islands, to name the most significant tax havens.^15^ The scale of tax evasion is difficult to assess, not least because the business model of tax havens has at its core a strict observance of bank secrecy. Gabriel Zucman estimates the global loss of tax revenue at around US $190 billion per year. French public finances in 2014 alone lost around a third of their potential wealth tax revenue due to tax havens.^16^

Much of the current literature on tax competition contains moral judgements that are somewhat unusual in the writing of academic economists. Gabriel Zucman conceptualises tax havens as thieves who steal tax revenues that are the rightful property of other states. Joel Slemrod and John D. Wilson go so far as to call tax havens “parasites”.^17^

Apart from the states which benefit from tax evasion, another focal point of the rapidly growing literature on tax evasion is individual tax evaders, the new super-rich. Several trade books with a strong popular appeal have attributed their behaviour to a changed relationship between individual and state. In this interpretation, billionaires become detached from specific societies and no longer feel any sense of responsibility for their development. Unlike the rich of earlier times, who presumably paid their taxes out of a sense of duty, this atomisation frees the new super-rich to maximise their financial interests by utilising every loophole and playing national governments against each other.^18^

Critics of both variations of the “perpetrator-victim-narrative” have argued that the states affected by tax evasion have not fully pursued strategies of international and supranational cooperation that could put a stop to tax evasion and money laundering practices. According to Andrea Binder, large economies like the US publicly complain about tax evasion, while undermining efforts for more transparency, like tax data exchange among OECD countries, by refusing to comply themselves.^19^ Big economies have also shown themselves reluctant to use access to their markets via licencing, import and travel restrictions as bargaining chips to ensure compliance with national or supranational fiscal regulations. In many cases, they have also failed to prosecute financial crimes to the full extent allowed by existing laws.^20^ Binder correctly points out that more research is necessary into what interest advanced economies might have in the continued existence of tax havens.^21^ Research on fiscal competition inside federal states indicates that governments which do not want to draw public attention to a *de facto* lower corporate tax rate, or lack the autonomy to change tax rates, accept tax evasion as a strategy to attract business.^22^

There is a consensus in the research literature that tax competition and tax evasion place limits on the ability of national governments to redistribute income and wealth. Both have incentivised governments to shift the burden on to labour and consumption to make up for lost or forgone revenue, to increase the public debt or to cut welfare payments. Most governments have applied all three strategies.^23^ However, there is a dispute among economists over whether a highly progressive tax regime is the best way to redistribute income. James Poterba argues that tax progressivity is not as effective as politically assumed, as it gives middle-class and working-class taxpayers a high incentive to avoid tax by changing their behaviour, and increases pre-tax inequality.^24^ Other economists, such as Stefan Svallfors and Eiji Yamamura, demonstrate that tax compliance is not just a one-dimensional reaction to taxation levels. Rather, they argue, the acceptance of taxation is directly connected to the degree of trust that social groups have in the government and in state institutions, and how they assess the quality of governance.^25^ Comparing the impact of redistribution in the US and European countries, Philipp Doerrenberg and Andreas Peichl theorise that the best government strategy would be to focus on a strong welfare state and view taxation as a means of financing it, rather than as an instrument of redistribution.^26^

### Educational Inequalities and Opportunity Hoarding

After World War II, numerous sociological studies drew attention to the lack of financial, logistical and other resources that hindered children from working-class and farming families from pursuing their education beyond the compulsory minimum. In most western societies, public debate treated such unequal educational opportunities as an issue of both social justice and economic development. Offering students from disadvantaged backgrounds the chance to fulfil their intellectual potential was meant to increase economic productivity by avoiding “educational wastage” and providing emerging industries with the required higher-skilled workforce.^27^

A common strategy to equalise educational opportunities was to open additional schools as well as universities and reduce or cut attendance fees. Together with the decreasing average number of children per family, these measures reduced the relative cost of prolonged education for parents. According to OECD-figures, the growing uptake of secondary and tertiary education caused the state expenditure on public education among member states to rise from 4.3 per cent in 1960 to 5.2 per cent of GDP in 1975. Within this general trend of educational expansion, significant national differences in public spending on schools and universities remained, varying from under 2 per cent in Greece to over 7 per cent in Denmark.^28^

Aside from a usually strong cross-partisan consensus for increasing public spending, education policy caused frequent conflicts between politicians of different ideologies and interest groups like parents’ organisations, teachers’ unions and employers.^29^ In much of Europe, the debate focussed on the merits of selective versus comprehensive school models and their respective effects on educational equality and quality. The founding of comprehensive schools was the most important strategy to equalise the abilities of children from different social backgrounds by exposing them to shared learning conditions for longer.^30^ The decision to ‘go comprehensive’ was often linked to the broader egalitarian reforms of social democratic governments during the 1960s and 1970s. Most prominently, Scandinavian countries implemented the comprehensive model to the fullest extent while also spending the highest proportion of GPD on public education. However, the development of education systems does not in all cases concur with the classic typology of welfare systems. The US, the prime example of a liberal market economy, also embraced a system of comprehensive schools and ranked close to the OECD mean in her spending on public education between 1960 and 2002. While public comprehensives, accompanied by a strong tradition of private, often religious schools and the legality of homeschooling, were relatively uncontroversial in the US, conflicts about public education here primarily concerned practices of racist segregation. During the mid-1960s, the American high school system provided 63 per cent of working-class children with a complete secondary education and, at least theoretically, the possibility to go to university. At the same time, only 3 per cent of English working-class children and only 1 per cent of their peers in Western Germany succeeded this far in their education.^31^ Both West Germany and the UK exemplify how public education became one of the policy areas with the most first-, second-and third-order policy changes.^32^ Both countries introduced comprehensive schools during the late 1960s and 1970s as a model for public schools and provided financial incentives for local authorities to implement it. The concept was altered significantly or abandoned during the 1980s by conservative governments. They also demonstrate a general trend of regional disparity since Scotland, Wales, as well as several (west‑)German federal states kept comprehensive schools while England and other parts of Germany retained or went back to a tripartite selective school system.

During the 1990s, a controversy ensued among educational sociologists about the effects of educational expansion and reforms on educational inequalities.^33^ A much-regarded meta-analysis of 13 European countries by Yossi Shavit and Hans-Peter Blossfeld and their controversial diagnosis of “Persistent Inequality” formed a critical reference point for this debate. According to Shavit/Blossfeld, the level of education had risen significantly across all classes, but children’s social background still determined the extent of their educational opportunities in most countries. Only Sweden and the Netherlands had managed to level the playing field for children from low-income families.^34^ The reaction to Shavit/Blossfeld’s study within the field of educational sociology has been mixed. The model of regression analysis that Shavit/Blossfeld use to measure education transitions (the “Mare model”) was challenged on methodological grounds as both simplistic and lacking statistical power.^35^ Several single-country studies also refuted the hypothesis of “Persistent Inequality”.^36^ However, other studies defended Shavit/Blossfeld’s findings based on national data and comparative analysis.^37^

A relatively rare but exemplary case, the Shavit/Blossfeld study has also been challenged by two replication studies with improved data sets. The first one of 2009 by Richard Breen and others reassessed the persistence-hypothesis based on more recent data and a larger sample from eight European countries.^38^ They used the ordered logit model to measure class inequalities via the class-specific odds of attaining five educational levels. Breen’s study covers five cohorts (males only) born between 1908 and 1964. Contrary to Shavit/Blossfeld, their findings substantiate the notion that democracies strove and mostly succeeded to close the class-based attainment gap. Children from working-class and farming families born between 1935 and 1954 benefitted the most from increased public spending on education.

However, there are some notable exceptions to this general trend of equalisation, even though Breen’s study only covers children whose educational career mainly occurred during times of high economic growth and expanding educational opportunities. In Western Germany, the class-based attainment gap continued to narrow for men born between 1925 and 1954 but stalled for the last cohort born between 1955 and 1964. This age group was first to pass through an expanded, more egalitarian education system, but the proportion of working-class children who received university-entrance qualifications only rose very slowly and stalled at around 10 per cent from the mid-1970s onwards.^39^ In Britain, the long-term trend of improvement for the sons of small business owners and skilled workers nearly ceased for the cohorts born after 1935. Some groups, such as sons of white-collar workers in France, did not experience any advancement over the entire post-war period. In Italy, class inequalities within the cohort born between 1955 and 1964 even grew more robust again.^40^

The second replication of Shavit/Blossfeld’s “Persistent Inequality”-study by Carlo Barone and Lucia Ruggera of 2018 covered an additional cohort born between 1965 and 1980 and educational careers between the 1940s and the 2000s. Their study is hence able to track the impact that numerous socioeconomic and political changes had on educational inequalities since the 1970s.^41^ Barone/Ruggera enlarge the cohort design regarding the number of countries (eight to 26) and the respective sample sizes. Unlike Breen’s study, they also examine both male and female educational careers. Barone/Ruggera’s findings confirm Breen’s refutation of the “Persistent Inequality”-hypothesis while addressing several methodological objections to the first replication study. They show a general long-term trend of statistically significant equalisation across five country clusters (Scandinavia, Western continental Europe including Israel, “Anglo-Saxon” Europe, Southern Europe, Eastern Europe) that proves robust for several calculation models. Despite having different education systems and spending levels, most countries reduced their kappa index, which measures the association between paternal social class and children’s educational attainment, between 40 and 50 per cent. Outliers are Sweden with 67 per cent and (Western) Germany as well as Great Britain with 17 per cent.^42^ Barone/Ruggera’s findings show that equalisation slowed or stalled for the last cohort born between 1965 and 1980 in most country clusters. Russia, Romania, Hungary and Bulgaria even display a curvilinear development: An initial flattening of educational inequality already started to reverse for students born after 1955 and hence significantly too early to be explained by the regime change in Eastern Europe.

Barone/Ruggera’s findings contribute to a survey-based narrative of how inequalities developed in Europe during and especially after the *trentes glorieuses*. However, the authors themselves point out that their approach is descriptive and cannot identify causal factors. Since the expansion of education systems and the reduction of monetary barriers to further education were necessary conditions for post-war equalisation, the most verisimilar explanation for its stalling would be the retrenchment of state financing. During the late 1970s, lack of economic growth and mass unemployment increasingly put pressure on public expenditure. Among OECD-member states, the mean proportion of GDP spent on education decreased from 5.9 per cent to 5.4 per cent (median from 6.1 to 5.3 per cent). Behind this overall retrenchment lay two different trajectories that led to a general trend of convergence. Countries that had spent the most on education between 1945 and 1975 also scaled back the most. The retrenchment group with all anglophone countries as well as the Netherlands, Norway, Finland and (Western) Germany comprises examples from all three types of welfare systems, which again speaks to the particular policy dynamics of public education. Manfred G. Schmidt has hypothesised a surprising link between spending on welfare and education in as much as countries like the Netherlands and (Western) Germany cut education budgets to maintain the structure of their welfare states under the pressures of mass unemployment.^43^ Unlike the high-spending retrenchment group, countries at the low end of the educational investment spectrum upped their spending on public education considerably after the mid-1970s. This trend was most robust in Spain, Portugal and Greece, where the idea of better educational opportunities for all merged with and was reinforced by the end of dictatorship and democratisation.^44^ These Southern European countries also displayed a continuous reduction of educational inequalities from the 1940s to the 2000s without the stalling effect common in other countries.^45^ Equalisation, in this case, occurred from an exceptionally high level of educational inequality, which responded strongly to the expansion of public education. Lower levels of educational inequality proved more resistant to state intervention even before education spending decreased relative to GPD.

A differential approach such as that of Erzsébet Bukodi and John H. Goldthorpe provides a valid explanation for the relative resistance of mid-level educational inequalities. They argue that much of the research on educational attainment only analyse one of the three components of social origin—social class (defined via economic capital), social status and parental education—leading to misinterpretations since they can develop incongruently.^46^ According to Robert Erikson/Bukodi/Goldthorpe’s subsequent comparative study on cohorts born in Sweden and the UK between the mid-1940s and the late 1960s, the effect of family finances on educational attainment remained stable. At the same time, the influence of parental social status declined, and that of parental education even increased.^47^ Barone/Ruggera disagree with their conclusions by arguing that parental education and social status combined proved the strongest predictors for educational attainment in the next generation. According to them, social class becomes statistically insignificant when controlled for these factors, with one intriguing exception, students from farming families.^48^ While the weighting between the components of social origin is in dispute, there is consensus that the importance of parental education relative to that of economic barriers increased over the post-war period. A comparative study into university education in France and England by Cécile Deer shows a similar persistence of inequalities despite a very different trajectory of public spending^49^: Since the mid-1970s, England cut spending on education, incrementally replaced maintenance grants for university students with loans and introduced up-front tuition fees in 1998. In contrast, France maintained her spending level and erected no such monetary barriers to higher education. However, access to university degrees—especially from highly prestigious institutions—remained primarily determined by social origin in both countries.

The following will briefly introduce two concepts from sociological and economic literature, that together can further explain the stalling of equalisation, educational assortative mating and opportunity hoarding.

Starting with the social upheavals caused by World War I, alternative visions of companionate marriage emerged alongside traditional ideas of marriage and started to grow mainstream in Western societies during the 1960s and 1970s*.*^50^ Part of the companionate ideal was a similarity in age and education between marriage partners. As a social phenomenon, educational homogamy rose between 1940 and 1970 when women started to close the educational attainment gap to men.^51^ During the 1960s, wives of high earners still earned less than the average woman, but the relationship between spouses’ earnings became positive from the 1970s onwards.^52^ When becoming parents, middle-class couples with dual university degrees often were willing to invest a significant amount of time and other resources into optimising their children’s education. They also were much more efficient at acquiring publicly financed educational resources for their children than parents with a smaller income and less education. Dawn Lyken-Segosebe and Serena E. Hinz have conceptualised such strategies as opportunity hoarding that often counteracted government policies intended to lower inequality.^53^ Most of the literature suggests that hoarding strategies are motivated by the idea that their children’s future social status is threatened by an increasing competition—whether real or imagined.^54^

Lyken-Segosebe/Hinz differentiate between within-school hoarding and between-school hoarding. Between-school hoarding aims at securing a place at the best possible local state school, either via official school choice or other means. Until the 1980s and 1990s, state schools in most countries recruited their students from a set catchment area, with parents officially having little or no choice. By the late nineteenth century, most cities had developed class-based and, in many cases, also ethnicity- or race-based neighbourhoods, often segregated by occupation or religion,^55^ which school catchment areas replicated.^56^ During the twentieth century, many such spaces of inequality were modified or reinforced by middle-class parents prioritising residences in the catchment areas of state schools that had a good reputation in their peer group.^57^ Contrary to what the predominantly sociological literature suggests, this was not a novel phenomenon of the 1980s. For the US, such strategies are well-documented ever since the inter-war period and, among other reasons, motivated the middle-class flight from the US inner cities to the suburbs. A study by John L. Rury and Argun Saatcioglu analyses the divide between the suburbs and inner cities based on the census and other serial data over a relatively long period, from 1940 to 1980.^58^

The clustering of the affluent classes in the suburbs had multiple repercussions. Local taxes in the suburbs had to support fewer welfare recipients so that more resources could go to the local schools. At the same time, ethnic minorities and working-class families who remained in the inner cities, or moved there for the lower rents, faced rising unemployment as well as poverty and sent their children to schools with much lower per-pupil budgets.^59^ Discriminatory zoning laws combined with high housing prices kept non-white working-class families out of the suburbs. This process of socioeconomic segregation between US suburbs and inner cities disrupted and reversed the slow narrowing of the ‘racial attainment gap’.^60^ Rury/Saatcioglu’s findings support the ‘Matthew principle’ that advantages accumulate for predominantly white, middle-class students in suburbia: affluence combined with relatively stable family life (at least in the US); a high degree of parental involvement; and a large proportion of successful state schools. By 1980 the ‘suburbia effect’ accounted for almost half of the higher attainment levels compared to pupils from inner-city schools. While concentration effects are a well-known phenomenon in poverty and deprivation,^61^ relatively little attention has been paid to their role in creating and reproducing wealthy milieus. The relevance of such evasion strategies depended, among other factors, on the fluidity of the housing market, which was high in the US case. In European cities like Athens, where residential segregation and housing market mobility were low, middle-class parents tended to opt-out of the state school system altogether by going private or tried to monopolise a local state school for children from their in-group.^62^ Selective state schools offered middle-class parents an alternative hoarding strategy without having to compete on the property market.

During the 1980s and 1990s, the demand for ‘freedom of choice’ in education gained momentum in most Western countries.^63^ Proponents of parental choice argued that this policy change created greater fairness by giving working-class parents an option that affluent parents had possessed all along.^64^ In many countries, catchment areas were abolished, and parents officially gained the right to choose schools for their children. In some cases, the educational choice debate also resulted in the provision of state funding for private schools.^65^ The change was particularly pronounced when combined with standardised testing and much public attention paid to rankings for schools and universities as in the UK and the US. Some studies claim that middle-class and upper-class parents primarily aim to avoid social and ethnic heterogeneity when choosing their children’s school, even above considerations of educational quality.^66^ However, in many countries, the market logic in public education remained limited, and school choice did not prove as averse to social equality as many social scientists had predicted.^67^

The new possibility to choose was also not universally welcomed by middle-class parents since it turned a high level of parental involvement from an option into a duty and the acceptance of state allocation into a form of neglect.^68^At least for some European countries, such as Germany and Finland, there is evidence that a segment of middle-class parents deliberately chose to send their children to the ‘regular’ local school. This decision depended on their assessment that their children did not risk being left behind.^69^ The education expansion had several unintended consequences which reinforced the perception of middle-class parents that their children’s future social status was under threat even though they proved highly effective in securing it. The slowdown of economic growth meant that the job market did not keep up pace with the uptake of higher education. This combination influenced the transition from the education system to the labour market on all levels. Not only did apprenticeships that used to be filled with applicants with mandatory minimum schooling increasingly attract students with university-entry qualifications. The relative inflation of credentials also meant that competition among college graduates increased, especially in the creative sectors of the economy. Middle-class parents often provided financial support so their children could meet new requirements like unpaid internships, extra-curricular activities and studies abroad.^70^ Working-class parents not only assessed the risk-benefit ratio of higher education differently than parents who were themselves college graduates, but they also had less insight into the rules of post-graduate labour markets and often lacked the resources invested by middle- and upper-class families.^71^

The concept of opportunity hoarding contributes to our understanding of how societies respond to attempts at social engineering and why universal welfare policies like public education can have unintended consequences that sometimes counteract policy goals. However, it remains unclear to what degree practising opportunity hoarding implies exclusionary intentions towards the groups that miss out on publicly financed resources as a result.

## Societal Impacts of Social Inequality

Over the last twenty years, a vast amount of research has examined the societal effects of economic inequality. Much of this debate was initiated and influenced by the work of social epidemiologists Kate Pickett and Richard G. Wilkinson. Their book “The Spirit Level” declared social inequality to be the determining factor in a long list of social problems. According to Pickett/Wilkinson, unequal societies were significantly more violent and unstable, with people suffering higher rates of illness and drug addiction. Their reasoning, in both “The Spirit Level” and their follow-up book, “The Inner Level”, was mainly social-psychological: the primary effect of inequality was to create anxiety and to disrupt trust in relationships, and this had multiple repercussions for both individual behaviour and societal cohesion. Similar to the strain theory, Pickett/Wilkinson saw social inequality as a structure that, in creating unachievable expectations for almost everyone except the rich, eroded their sense of self-worth.^72^

Published in 2009, when most countries were suffering the full impact of the financial crisis, “The Spirit Level” hit a nerve, especially with the British and US public, and became a bestseller despite its extensive—and controversial—use of statistics.^73^ Pickett/Wilkinson offered an apparently easy fix for almost everything that was wrong with contemporary society, and also provided a potential reason for the rich to act contrary to their own economic interests. If redistributive policies could make society more peaceful and everyone happier, the rich, too, would benefit directly from a higher quality of life, and not just in a common good or altruistic sense. The following section will examine how the spirit-level hypothesis stands up to the research literature on three important societal impacts of growing social inequality: crime, happiness and openness.

### Crime

The effects of social structure on a society’s crime levels are a long-standing research focus of both sociology and criminology. The theory of *social disorganisation*, which has mostly dominated this field of research since the 1920s, attributes crime to a combination of poverty and a lack of social interconnectedness, since both impede a community’s ability to enforce shared values. It signified a fundamental shift in the mainstream interpretation of deviant behaviour, moving from individual- or race-based causation to an environmentally based explanation. Following the Chicago School approach, most studies focused on the neighbourhood level of big cities and compared the crime rates of areas with different income levels. Current literature in the field of social criminology considers both poverty and deprivation relative to adjacent neighbourhoods. It has shown inequality to be the more powerful explanatory variable for rising crime rates.^74^ A long-standing and contested issue in this literature concerns the different effects an increase in income inequality has on property crimes and on violent crimes against people.^75^ Current research also examines the effect of rising income inequality in different countries from a comparative perspective. The hypothesis of a positive causal link between inequality and crime rates has long been considered a ‘western theory’ that only applies to industrialised countries. However, research on China shows that inequality in income, employment and consumption positively correlates with different levels of crime between Chinese regions.^76^

Cross-country comparisons face several methodological issues, not all of which are reflected in the criminological and sociological literature. Most of these comparisons use highly aggregated time series data to test the robustness of correlations between some variant of Gini-Index and violent crime rates relative to population size versus other influences.^77^ Time series data are more reliable than sampling individual years as a basis of comparison. However, they must be analysed as source material that is a product of changing circumstances and perceptions. Since the 1960s, definitions of crimes and types of prosecution have changed significantly in many countries. These changes are most apparent when it comes to crimes against an individual’s sexual self-determination and domestic violence.^78^ Similar problems apply to synchronic comparison because of differences in legal systems.

On the whole, the hypothesis that societies with comparatively high inequality are also more violent is broadly accepted in the fields of both criminology and sociology. However, the example of crime also shows that attempts to use this insight as a basis for social engineering have produced unintended consequences. During the late 1990s and early 2000s, several countries added analytical tools to their legal procedures that automatically factored socioeconomic deprivation into sentencing procedures. Remarkably, the outcomes of such attempts were similar in countries with different levels of social inequality (Canada, US, UK and the Netherlands): these analytical tools turned economic deprivation into a high-risk factor for re-offending behaviour. Such coding resulted in longer sentencing and lower chances for indigent defendants to be released on probation instead of going to prison when compared with their more affluent counterparts. Rather than making justice systems more equal, the automated consideration of social inequality reinforced existing similarity bias in favour of middle- and upper-class defendants.^79^

### Happiness

While the spirit-level hypothesis by and large holds up in relation to crime, the research literature on the link between inequality and happiness is much more ambiguous. Mostly due to methodological differences, there is currently no consensus in the research fields of sociology, economics and social psychology on the question of whether unequal societies are more unhappy or prone to depression than those with less discrepancies in income distribution.^80^ Similar disagreement exists about the capacity of government interventions like redistributive taxation and welfare payments to alter a society’s happiness level.^81^

Most research literature conceptualises happiness as one component of individual well-being (with overall life satisfaction being the other one) and relies on self-assessments to measure it.^82^ Since the 1970s, happiness economics has aimed to answer the question of how the growing prosperity that industrialised countries experienced after the Second World War affected the mood of their citizens. The so-called Easterlin paradox has dominated this field—and popular perceptions of happiness economics—as the result of a pioneering study based on US data from 1974 onwards. At the core of this paradox is the finding that in the long-term happiness does not increase in line with disposable income beyond a point of satiation.^83^ The Easterlin paradox has been challenged on the basis of comparative data but still provides a focal point for debate within the field of happiness economics. Since the Easterlin paradox seems to refute the idea that GDP is an explanatory factor for aggregate happiness, a wide range of studies have considered the unequal distribution of growing prosperity to be decisive.^84^

Apart from aggregated happiness levels, some studies in the field of happiness economics utilise the concept of happiness inequality, measuring the level of happiness across and within different social and ethnic groups. One of the best sources for the development of happiness inequality is the “US General Social Survey”. It has been asking a representative sample of Americans to rate their happiness from low to moderate to high continuously since 1972.

Most research on happiness inequality does not refute the Easterlin paradox, but complements it with two valuable insights. First, there was an overall trend towards moderate happiness during the 1970s and 1980s (rising from 49 per cent in 1972 to 58 per cent in 1991), while the share of Americans who reported that they were “not too happy” fell from 14 per cent in 1972 to its lowest level of under 8 per cent in 1990. While the proportion of participants claiming a high degree of happiness has been on a downward trend since the early 1970s, they still outnumber those with low happiness by 3 to 1.^85^ The rapid growth in social inequality has not led to greater happiness inequality among the US population overall.

Second, the “General Social Survey” data demonstrates a general trend towards cross-group convergence since the 1970s. The gap between men and women has almost disappeared, with women reporting decreasing levels of happiness while men feel significantly better about their lives. A similar trend towards convergence has occurred between whites and non-whites, but the gap was much bigger than the gender gap in the 1970s and was still more pronounced during the 2010s. One exception to this general narrative of convergence is notable. The happiness gap between university graduates and individuals without a completed secondary education has widened, presumably because of an increased risk of unemployment for high school dropouts and the increasing financial return on higher education over the life cycle.^86^ Several studies have confirmed the detrimental effect of unemployment on happiness levels. A study comparing Europe and the US argues that unemployment and not income inequality is the socioeconomic factor that determines happiness.^87^

Several US surveys show that the effects of income inequality on happiness depend not only on objective circumstances, but also on individual psychological factors like personality. Respondents from all income groups who attached great importance to how their income compared to that of others were less satisfied with their life than social peers who did not.^88^ This research literature also demonstrates the role of local social comparisons: people tend to judge their economic status against that of face-to-face contacts instead of abstract concepts such as national averages. Family members of the same gender with whom there is a climate of competition are of crucial importance, corroborating H. L. Mencken’s quip that for a man, being wealthy means earning significantly more than his brother-in-law.^89^ A 2019 study based on data from the “European Quality of Life Survey” demonstrates that social comparisons have specific group effects in addition to the individual coping with social inequality. This European data confirms the importance of close social contacts as a reference point, since in-group inequality decreases happiness at all levels of income, while growing inequality between groups shows no effect. In addition to such group effects, the study also traces the adverse effect of inequality on all income groups.^90^

The research literature on happiness economics, on the whole, shows little or no reflection on how social and cultural contexts shape individual assessments of happiness. A historical-critical secondary analysis of the extensive survey data that is available for the post-1970s period could enrich this field by pointing out the influence both of socioeconomic changes as well as perceptions and expectations within social spaces like milieus.^91^

### Societal Openness: The Politics of Resentment and Welfare Chauvinism

The rise of a new right-wing populism since the 1990s has sparked a debate about whether growing social inequality made societies more exclusionary or even resentful towards those considered alien to a polity defined in racist or nativist terms.

Ironically, it was political scientist Francis Fukuyama who has made one of the most influential contributions to this debate on the crisis of liberalism and universalism.^92^ In his book “Identity” (2018), Fukuyama argues that the social inequality brought about by globalisation was a necessary condition for the rise of populism and aggressive identity politics but cannot explain it in and of itself. Instead, he suggests, socioeconomic changes like deindustrialisation triggered feelings of being disrespected and ignored by the media and political decision-makers alike that turned the loss of income and status into long-lasting grievances. Self-styled anti-establishment elites used these resentments to mobilise voters against a variety of targets, including minorities. Fukuyama is arguing against the simplistic idea of humans as rational maximisers of economic interests, which, he claims, dominates public discourse, and for the importance of emotions in politics.^93^ His book certainly offers some interesting insights into how the politics of resentment might work. It appeals especially to politicians and political commentators who perpetuate the narrative that recent triumphs of populism were the revenge of those left behind in economic terms. However, Fukuyama’s argument lacks both empirical evidence and analytical clarity. An analysis of emotions must amount to more than speculating that everyone who expresses resentment towards minorities or multiculturalism does so because of presumed experiences of economic loss and humiliation.^94^

Empirical studies offer little support for the assumption that right-wing populist parties (RRPs) are especially appealing for voters in precarious economic situations or represent their interests. The new RRPs did not define themselves as pro-welfare or pro-redistribution parties. At least in their early phase, in the late 1980s and 1990s, most took a neoliberal approach to economic and social policy.^95^ The concept of *welfare chauvinism*, which was adopted by almost all European RRPs during the 1990s, demanded that only in-group members should be entitled to welfare payments and only under certain conditions. By racialising the old idea of the deserving poor, similar to the Nazi *Volksgemeinschaft*, welfare chauvinism offered RRPs the opportunity to reject or even demonise the universal welfare state and still claim to represent those who had lost out due to globalisation and other “globalist elite” projects.^96^

A frequently cited article by sociologist Daniel Oesch looks at class-related differences in right-wing attitudes in France, Belgium, Switzerland, Austria and Norway. In all five cases, working-class voters were more likely than middle-class voters to choose RRPs. This gap varied between 300 per cent in Belgium/France and 130 per cent in the Netherlands. Oesch operationalises economic reasons, such as insecure employment and fear of wage pressures, to explain this voting behaviour along the lines of the ethnic competition hypothesis. Differential nativism and disenchantment with the political system could coincide with economic deprivation or the perceived risk of it. However, most working-class supporters of RRPs were “insiders”, skilled workers with steady jobs.^97^ Oesch argues that cultural fears about immigration as a threat to national identity offer the most robust explanation for their support of RRPs. Oesch’s assumption that workers might reject multiculturalism because they have “fewer cognitive skills” smacks of classism.^98^ A historical perspective on the development and decline of an original working-class identity, accompanied by the universalisation of a middle-class lifestyle that was financially unachievable for most manual workers, offers a better explanation for cultural anxieties among this group.^99^ According to the current political science literature, the differing attitudes to multiculturalism of working-class and middle-class voters are part of the dilemma that social democratic parties have faced over recent decades. Along with differing demands of welfare reform, this electoral cleavage has restrained many European centre-left parties from turning rising social inequality into an election-winning issue.^100^

German survey data from the 1980s and 1990s suggests that nativism among the unemployed and those with precarious jobs—the people most likely to be affected by ethnic competition—did not equal a permanent resentment of immigration. Rather, Marcel Coenders and Peer Scheepers argue, their exclusionist attitudes were a temporary reaction to recent increases in unemployment and immigration. Neither of these disadvantaged groups displayed a lasting orientation towards right-wing populism.^101^ Studies with a comparative European approach reach a similar conclusion: neither the level of unemployment nor the proportion of precarious employment could explain the differing prevalence of nativism and racist attitudes.^102^

The acrimonious Brexit campaign and the electorate’s narrow (52 to 48 per cent) decision to leave the European Union has startled many commentators and resulted in a major political victory for right-wing populist strategies of mobilisation. In a particularly intriguing paper of 2020, economists Maria Abreu and Özge Öner have analysed how different socioeconomic, political and cultural factors impacted the likelihood of constituencies to vote to leave the European Union.^103^ One of Abreu/Öner’s findings points to a particularity of the Brexit campaign: Constituencies with a low voter turnout in previous elections were more likely (3.1 percentage points) to vote leave than others. This success for the leave campaign is likely down to a novel type of micro-targeted advertising on social media that used a combination of psychographics and big data to influence or nudge previous non-voters with an alleged anti-establishment message to participate in a protest vote.^104^

Abreu/Öner’s analysis supports the hypothesis that “values-based cultural grievance(s)” were more influential in the Brexit vote than socioeconomic circumstances like inequality. Amongst economic variables, only low wage growth was more prevalent in pro-Brexit- than in pro-remain-constituencies. Given that Brexit has mostly been interpreted as a working-class vote against globalisation, this difference with 1.8 percentage points is much less significant than expected. As Jim Tomlinson argues in his fascinating book “Managing the Economy, Managing the People. Narratives of Economic Life in Britain from Beveridge to Brexit of 2017”, seeing Brexit as an anti-globalisation revolt accords with a dominant public discourse by politicians and journalists that depicted globalisation as both inevitable and ubiquitous while giving little to no mention to benefits like cheap imported consumer goods. There is little doubt that wage stagnation and wage polarisation increased social inequality in the UK and drove resentment against the economic status quo, which many voters identified with the EU. However, most of the British wage development is due to long-standing processes of deindustrialisation in which globalisation only played a relatively recent, cumulative role.^105^ Tomlinson also points out that immigration and a Europeanised labour market turned out the most problematic topic for the remain campaign.^106^

Abreu/Öner’s data supports this argument: Anti-immigration attitudes with seven percentage points were a much more decisive factor than low wage growth to tip the majority against a continued EU membership and the freedom of movement that goes along with it. Of particular interest is that Abreu and Öner can show the difference between how immigration is perceived (presumably through the media) and how it is experienced in everyday life in the local community. Contrary to the hypothesis of ethnic competition, economically successful constituencies with low immigration levels were more likely to reject European immigration than economically slow-growing areas with high levels of immigration.^107^ The leave campaign’s strategy of (falsely) suggesting that Turkey would join the EU imminently with millions of Turkish citizens coming to the UK proved an effective mobilisation tool.^108^

There are several similarities and entanglements between the Brexit vote and the other recent major success for right-wing populism, the victory of Republican candidate Donald Trump over Democratic candidate Hillary Rodham Clinton in the 2016 US presidential election. Much of the political commentary has reproduced the claim of self-styled ‘blue-collar billionaire’ Donald Trump to represent a revolt of downwardly mobile workers and middle-class voters in difficult financial situations. The research literature is more mixed, with some scholars pointing to the low education level of the average Trump voter and reviving Lipset’s idea of ‘working-class authoritarianism’ while others dispute the notion.^109^ Trump won around 62 per cent of the white working-class vote, which is a level similar to Richard Nixon’s election of 1972.^110^ When looking at a voter breakdown along income groups instead of class categorisations, it becomes clear that Trump especially appealed to voters with mid-level and higher incomes. Hillary Clinton received an absolute majority among voters with very low (under 30,000$) and very high (over 120,000$) incomes.^111^ The median annual income of Trump voters surpassed the state-wide median income in every state (gap ranging from 2,000 to 29,000$) and the national median income by more than 10,000$.^112^

Data from the “American National Election Studies”—a survey that has been continuously conducted on presidential elections since 1948—again demonstrates how crucial cultural grievances are for the electoral appeal of right-wing populism. Negative attitudes towards minorities and feminism combined with preferences for authoritarian leaders were the best predictor of who would vote for Trump.^113^ Trump’s electoral success was mostly due to his ability to mobilise more radical nativist voters and simultaneously maintain the support of traditional conservatives while Clinton failed to motivate the so-called Obama coalition to turn out in sufficient numbers for her, especially in the mid-west.^114^

Much of the cited research pits socioeconomic explanations of electoral behaviour against theories that focus on cultural interpretations of belonging and resentment. In their thought-provoking article “Three Worlds of Welfare Chauvinism”, Jeroen Van Der Waal, Willem De Koster and Wim Van Oorschot argue that neither cultural nor economic theories alone can explain why exclusionary tendencies between countries differ. Their analysis of data from the “European Social Survey” for 2008 combined with country-level census data points to the importance of institutional settings and path dependency. Der Waal/De Koster/Van show that countries with strong welfare states—the social-democratic type in Gøsta Esping-Andersen’s well-known model—display the lowest level of welfare chauvinism. The authors hypothesise that the resulting lower social inequality led to less divergent lifestyles between different income groups. They argue that relative similarity fostered an understanding towards low-income groups and prevented stigmatisation—an effect that extended to welfare recipients and immigrants.^115^ An article by Jonas Edlund and Arvid Lindh also confirms the welfare state inequality hypothesis by a comparative analysis of data from the “International Social Survey Program” for 20 countries and additional data. However, their reasoning is somewhat different. Unlike Van Der Waal/De Koster/Van Oorschot, who emphasise cross-class harmony, Edlund/Lindh link the lower level of welfare chauvinism in countries with effective redistributional policies to Walter Korpi’s theory of class conflict.^116^ This interpretation suggests that strong welfare states channel social conflicts into political conflicts that are expressed and regulated through institutions.^117^

While both articles present convincing arguments for the welfare state-inequality-hypothesis, they also demonstrate the need for further research. A longer-term comparative perspective that links the development of welfare states and social inequality with an analysis of exclusionary attitudes and resentment towards minorities would help determine the role of individualised live risks in societal processes of inclusion and exclusion.

## The Issue of Long-Term Social Mobility: Nature Versus Nurture Reloaded

In 2014, economist Gregory Clark published a widely noticed book that challenged the consensus on social mobility by proposing the existence of a “universal constant of intergenerational correlation of 0.75, from which deviations are rare”.^118^ Clark and his team examined a global range of case studies (drawn, among others, from China, India, Chile, Sweden, and England) in a long-term perspective from the Middle Ages to the twenty-first century, following the socioeconomic status of families with unusual—and hence traceable—surnames. The apparent differences between these societies, their historical circumstances, cultures and level of government intervention make Clark’s hypothesis of a uniformly (low) level of social mobility even more astonishing.

Unlike studies that take into account only how individuals fare relative to their parents (mostly the father), Clark applies a multigenerational approach, and he credits this as part of the reason why their estimates of social mobility are much higher than his. Families with Norman surnames, for example, provide impressive evidence for the long-term preservation of social status. Their huge overrepresentation among Oxbridge graduates during the twelfth century declined gradually over time but was still visible in the late twentieth century. Clark’s argument for a multigenerational research design is convincing, but it is not as unique as the author claims, given that the so-called grandparents’ effect is well documented in the literature on social inequality.^119^ Grandparents tend to play a significant role in reproducing their socioeconomic status in their grandchildren’s employment history.^120^ This seems especially plausible for the US and Western European cohort that was born during the 1930s: they reached their maximum earning capacity during an economic boom phase, bought houses in record numbers and paid off their mortgages during the 1980s.^121^ Being free of this debt released economic capital just at the time when their grandchildren were reaching school age, allowing for intergenerational investment and adding to several other dynamics that increased social inequality during the 1980s.^122^

While Clark’s idea of following families with low-probability surnames over a long period is a considerable innovation, there are several grounds for serious critique. The first is methodological: the categories he uses to describe socioeconomic positions are ‘elite’ and ‘underclass’, which lack a clear definition based on income, property, and other forms of capital. A long-term perspective intensifies the analytical weaknesses of such vague categories. The suggestion that graduating from prestigious universities like Oxford in the thirteenth century or Harvard in the seventeenth transferred the same kind of elite position as in the twentieth century makes this research design practicable.^123^ However, the lack of historical context diminishes its explanatory power, especially since the declared goal is not to examine elites in and of themselves, but to compare the development of relational positions within specific societies. Another methodological issue concerns how Clark arrives at his “universal constant”. As sociologist Ineke Maas has pointed out, Clark’s data does not necessarily lead to his conclusion of cross-case and cross-temporal uniformity. To do so requires substantial corrections for migration flows, for example, that are applied in some case studies but not in others.^124^

The second ground for critique relates to the far-reaching conclusions Clark draws from his findings, especially his contribution to the nature-nurture debate. He takes a position close to genetic determinism on the spectrum of this debate, arguing that some families are successful in the long term because they pass on superior abilities to their offspring via their genes. There are several objections to equating status and genetic inheritance as Clark does. Families, as documented by surnames, are primarily defined by law via the presumption of paternity, not necessarily by genetics, and their gene pool is increasingly diversified with every generation. Clark goes even further by claiming that social inequalities are fair and ‘natural’, the result of unequally distributed “innate talent” that is mostly resistant to intervention.^125^ This conclusion seems like a dusty souvenir of the highly ideological nature-nurture debate of the 1980s and 1990s. In the US in particular, this debate was conducted by both social scientists and natural scientists, and quite openly formed part of a political argument about the role of government in changing unequal access to resources. Without much support from scientific evidence for such a simplistic interpretation, the political ‘nature’ advocates questioned the prudence of investing in policies designed to produce more social mobility if genetics had already determined an individual’s abilities. This discourse culminated in the book “The Bell Curve”, published in 1994,^126^ and the social Darwinist—and in some cases openly racist—positions that drew legitimacy from it. These interpretations of social inequality helped to move the US political mainstream away from the New Deal consensus and enabled the fundamental changes to the welfare state during the Clinton presidency.^127^ Both Herrnstein/Murray’s “Bell Curve” and Clark’s “The Son also Rises” demonstrate the often complex interdependence between scientific output and dominant interpretations of social realities within the political field. Future research on social inequality will benefit from the political relevance attributed to its subject in terms of visibility and access to additional funding. However, this role poses additional challenges to reflect not only on how societal interests and expectations shape research. Scientific communities also need to agree on best-practice strategies to prevent cherry picking, oversimplification and instrumentalization when transferring their results into public debate.

## Conclusion

This article aims to provide an interdisciplinary review of research literature on social inequality. It focuses on three main issues that have inspired much debate in economics, sociology, political science and history.

The first concerns the ability of governments to redistribute income and wealth under the pressures of globalisation, and the tendency of modern societies to resist governance. This part of the article follows the debates about two research concepts, tax competition and opportunity hoarding. The literature demonstrates numerous limitations and unintended consequences of government intervention, but also points to a lack of political will to implement redistributional policies effectively.

The second issue under review is the impact that growing social inequality had on the development of Western societies from the 1970s. Taking Pickett/Wilkinson’s ‘spirit level’ hypothesis as a point of departure, the article reviews the literature on crime, happiness and societal openness. While growing social inequality, on the whole, corresponds to increasing crime levels in most societies, the effects on happiness are less clear. Finally, exclusionary tendencies showed a robust correlation both with social inequality and the redistributional aims of the welfare state.

The third issue concerns the influence of genetics on long-term trends in social mobility. This part of the article reviews Clark’s hypothesis that social mobility follows a universal constant because of the ‘natural’ distribution of abilities within societies and points out several methodological issues with his approach.

## Auswahlbibliografie

- Abreu, Maria/Öner, Özge: Disentangling the Brexit Vote. The Role of Economic, Social and Cultural Contexts in Explaining the UK’s EU Referendum Vote, in: Environment and Planning A. Economy and Space 52 (2020), No. 7, pp. 1434–1456.
- Barone, Carlo/Ruggera, Lucia: Educational Equalization Stalled? Trends in Inequality of Educational Opportunity between 1930 and 1980 across 26 European Nations, in: European Societies 20 (2018), No. 1, pp. 1–25.
- Blank, Grant/Graham, Mark/Calvino, Claudio: Local Geographies of Digital Inequality, in: Social Science Computer Review 36 (2018), No. 1, pp. 82–102.
- Lyken-Segosebe, Dawn/Hinz, Serena E.: The Politics of Parental Involvement. How Opportunity Hoarding and Prying Shape Educational Opportunity, in: Peabody Journal of Education 90 (2015), No. 1, pp. 93–112.
- Piketty, Thomas: Capital and Ideology, Harvard UP, Cambridge, MA/London 2020.
- Wilkinson, Richard/Pickett, Kate: The Inner Level. How More Equal Societies Reduce Stress, Restore Sanity and Improve Everyone’s Wellbeing, Penguin, London 2018.

## Footnotes

1 HBO: “Last Week Tonight with John Oliver”, Season 1, Episode 10, 13 July 2014.

2 Piketty, Thomas: Capital in the Twenty-First Century, Harvard UP, Cambridge, MA/London 2014.

3 See as an example the public dispute between Thomas Piketty and the economics editor of the “Financial Times” Chris Giles: Giles, Chris/Giugliano, Ferdinando: Thomas Piketty’s Exhaustive Inequality Data Turn out to be Flawed, in: Financial Times, 23 May 2014, URL: <https://on.ft.com/2SBs3aA> [accessed: 04.10.2020].

4 Tanzi, Vito/Schuknecht, Ludger: The Growth of Government and the Reform of the State in Industrial Countries, in: Solimano, Andre (ed.): Social Inequality. Values, Growth, and the State, Michigan UP, Ann Arbor, MI 1998, pp. 171–207. For the state expansion before World War II, see Lindert, Peter: The Rise of Social Spending, 1880–1930, in: Explorations in Economic History 31 (1994), No. 1, pp. 1–37; Aidt, Toke S. et al.: Democracy Comes to Europe. Franchise Extension and Fiscal Outcomes 1830–1938, in: European Economic Review 50 (2006), No. 2, pp. 249–283.

5 For an interesting analysis that questions the usual periodisation of welfare state development see Wincott, Daniel: The (Golden) Age of the Welfare State. Interrogating a Conventional Wisdom, in: Public Administration 91 (2013), No. 4, pp. 806–822.

6 Schäfer, Armin: Krisentheorien der Demokratie. Unregierbarkeit, Spätkapitalismus und Postdemokratie, MPIfG discussion paper No. 08/10, Cologne 2008, URL: <https://www.mpifg.de/pu/mpifg_dp/dp08-10.pdf> [accessed: 29.10.2021]; Birch, Anthony H.: Overload, Ungovernability and Delegitimation. The Theories and the British Case, in: British Journal of Political Science 14 (1984), No. 2, pp. 135–160.

7 See for this debate Cerny, Philip: International Finance and the Erosion of State Policy Capacity, in: Gummet, Philip (ed.): Globalisation and Public Policy, Elgar, Cheltenham 1996, pp. 83–104.

8 Scheidel, Walter: The Great Leveler. Violence and the History of Inequality from the Stone Age to the Twenty-First Century, Princeton UP, Princeton, NJ/Oxford 2017.

9 Piketty, Thomas: Capital and Ideology, Harvard UP, Cambridge, MA/London 2020.

10 Paidipaty, Poornima/Pinto, Pedro Ramos: Revisiting the “Great Levelling”. The Limits of Piketty’s *Capital and Ideology* for Understanding the Rise of Late 20th Century Inequality, in: British Journal of Sociology 72 (2021), No. 1, pp. 52–68.

11 As Marc Buggeln has pointed out, during the 1960s the anticipation of tax evasion already caused some countries—especially in southern Europe—to rely heavily on consumption taxes for revenue with adverse effects on the development of social equality. See Buggeln, Marc: Taxation in the 1980s. A Five-Country Comparison of Neo-Liberalism and Path Dependency, in: Buggeln, Marc/Daunton, Martin/Nützenadel, Alexander (eds.): The Political Economy of Public Finance. Taxation, State Spending and Debt since the 1970s, Cambridge UP, Cambridge 2017, pp. 105–125.

12 Genschel, Philipp/Schwarz, Peter: Tax Competition. A Literature Review, in: Socio-Economic Review 9 (2011), No 2, pp. 339–370, here p. 340.

13 Ibid., pp. 348f.

14 See for the historical development of tax havens Farquet, Christophe: The Swiss Tax Haven, the Bretton Woods System Crisis and the Globalisation of Offshore Finance, in: Buggeln, Marc/Daunton, Martin/Nützenadel, Alexander (eds.): The Political Economy of Public Finance. Taxation, State Spending and Debt since the 1970s, Cambridge UP, Cambridge 2017, pp. 126–148.

15 Winner, Hannes: Has Tax Competition Emerged in OECD Countries? Evidence from Panel Data, in: International Tax & Public Finance 12 (2005), pp. 667–687.

16 Zucman, Gabriel: The Hidden Wealth of Nations. The Scourge of Tax Havens, Chicago UP, Chicago, IL 2015, p. 50.

17 Slemrod, Joel/Wilson, John D.: Tax Competition with Parasitic Tax Havens, in: Journal of Public Economics 93 (2009), No. 11–12, pp. 1261–1270.

18 See as examples Zitelmann, Rainer: The Wealth Elite. A Groundbreaking Study of the Psychology of the Super Rich, LID, London 2019; Luzkow, Jack Lawrence: Monopoly Restored. How the Super-Rich Robbed Main Street, Palgrave Macmillan, Basingstoke/New York 2018; Birtchnell, Thomas/Caletrío, Javier: Elite Mobilities, Routledge, London 2014; Irvin, George: Super Rich. The Rise of Inequality in Britain and the United States, Polity, Cambridge 2008; Haseler, Stephen: Super-Rich. The Unjust New World of Global Capitalism, Palgrave Macmillan, Basingstoke/New York 2000.

19 Binder, Andrea: The Hidden Wealth of Nations. The Scourge of Tax Havens, in: Cambridge Review of International Affairs 29 (2016), No. 3, pp. 1186–1188.

20 See for examples Buchanan, Bonnie/Zabala, Craig Anthony: Money Laundering and Legal Compliance in the US Financial Services Industry. The Case of Standard Chartered Bank, in: Aßländer, Michael/Hudson, Sarah (eds.): The Handbook of Business and Corruption. Cross-Sectoral Experiences, Emerald, Bingley 2017, pp. 255–278; Chaikin, David: Money Laundering and Tax Evasion. The Assisting of the Banking Sector, in: Aßländer, Michael/Hudson, Sarah (eds.): The Handbook of Business and Corruption. Cross-Sectoral Experiences, Emerald, Bingley 2017, pp. 237–254.

21 Binder: Wealth (see footnote 19).

22 See as examples Stöwhase, Sven/Traxler, Christian: Tax Evasion and Auditing in a Federal Economy, in: International Tax and Public Finance 12 (2005), No. 4, pp. 515–531; Cremer, Helmuth/Gahvari, Firouz: Tax Evasion, Fiscal Competition and Economic Integration, in: European Economic Review 44 (2000) No. 9, pp. 1633–1657.

23 The burden on labour has increased mostly due to higher social security contributions. See Genschel, Philipp: Globalisation, Tax Competition, and the Welfare State, in: Politics & Society 30 (2002), No. 2, pp. 245–275, here p. 261.

24 Poterba, James M.: Income Inequality and Income Taxation, in: Journal of Policy Modeling 29 (2007), No. 4, pp. 623–633.

25 See Yamamura, Eiji: Trust in Government and its Effect on Preferences for Income Redistribution and Perceived Tax Burden, in: Economics of Governance 15 (2014), No. 1, pp. 71–100; Svallfors, Stefan: Government Quality, Egalitarianism, and Attitudes to Taxes and Social Spending. A European Comparison, in: European Political Science Review 5 (2013), No. 3, pp. 363–380.

26 Doerrenberg, Philipp/Peichl, Andreas: The Impact of Redistributive Policies on Inequality in OECD Countries, in: Applied Economics 46 (2014), No. 17, pp. 2066–2086.

27 Kafka, Judith: Inequality in Education, in: Rury, John L./Tamura, Eileen H. (eds.): The Oxford Handbook of the History of Education. Oxford UP, Oxford et al. 2019, pp. 334–354.

28 See for all cited data on public education spending Schmidt, Manfred G.: Testing the Retrenchment Hypothesis. Educational Spending 1960–2002, in: Castles, Francis G. (ed.): The Disappearing State? Retrenchment Realities in an Age of Globalisation, Elgar, Cheltenham 2007, pp. 159–183, here p. 160.

29 See for a both theoretical and empirical fruitful cross-country analysis of the nexus between educational settings, labour markets and social inequality Busemeyer, Marius R.: Skills and Inequality. Partisan Politics and the Political Economy of Education Reforms in Western Welfare States, Cambridge UP, Cambridge 2014.

30 Barone, Carlo/Ruggera, Lucia: Educational Equalization Stalled? Trends in Inequality of Educational Opportunity between 1930 and 1980 across 26 European Nations, in: European Societies 20 (2018), No. 1, pp. 1–25, here p. 6.

31 Kafka: Inequality (see footnote 27), p. 345.

32 See for the concept of a hierarchy between policy changes Hall, Peter A.: Policy Paradigms, Social Learning, and the State. The Case of Economic Policymaking in Britain, in: Comparative Politics 25 (1993), No. 3, pp. 275–296.

33 See for an overview of sociological research on the intergenerational transmission of socioeconomic advantages Ganzeboom, Harry et al.: Comparative Intergenerational Stratification Research. Three Generations and Beyond, in: Annual Review of Sociology 17 (1991), No. 1, pp. 277–302.

34 Shavit, Yossi/Blossfeld, Hans-Peter (eds.): Persistent Inequality. Changing Educational Attainment in Thirteen Countries, Westview, Boulder, CO 1993.

35 See for the criticism of oversimplification Lawton, Denis: Reviewed Work(s): *Changing Educational Attainment in Thirteen Countries* by Y. Shavit and H. P. Blossfeld, in: British Journal of Educational Studies 42 (1994), No. 4, pp. 413–415. For a positive evaluation of the “Mare model” see Blossfeld, Pia N.: A Note on the Mare Model (Arbeitsbericht des Insituts für Soziologie Nr. 76), Institut für Soziologie der Universität Leipzig, Leipzig 2018, URL: <https://core.ac.uk/download/pdf/226129336.pdf> [accessed: 27.10.2021].

36 See as examples Jonsson, Jan O et al.: A Half Century of Increasing Educational Openness? Social Class, Gender and Educational Attainment in Sweden, Germany and Britain, in: Erikson, Robert/Jonsson, Jan O. (eds): Can Education Be Equalized? The Swedish Case Comparative Perspective, Westview, Boulder, CO 1996, pp. 183–206; Ballarino, Gabriele [et al.]: Persistent Inequalities? Expansion of Education and Class Inequality in Italy and Spain, in: European Sociological Review 25 (200), No. 1, pp. 123–138; Kivinen, Osmo/Rinne, Risto: Higher Education, Mobility and Inequality. The Finnish Case, in: European Journal of Education 31 (1996), No. 3, pp. 289–310.

37 See as examples Pfeffer, Fabian T.: Persistent Inequality in Educational Attainment and its Institutional Context, in: European Sociological Review 24 (2008), No. 5, pp. 543–565; Herz, Tom et al.: The Inheritance of Educational Inequality. International Comparisons and Fifty-Year Trends, in: The B.E. Journal of Economic Analysis & Policy 7 (2007), No. 2, pp. 163–180; Hout, Michael/Janus, Alexander: Educational Mobility in the United State Since the 1930s, in: Duncan, Greg J./Murnane, Richard J. (eds.): Whither Opportunity? Rising Inequality, Schools, and Children’s Life Chances, Russell Sage, New York 2011, pp. 165–185.

38 Breen, Richard: Nonpersistent Inequality in Educational Attainment. Evidence from Eight European Countries, in: American Journal of Sociology 114 (2009), No. 5, pp. 1475–1521.

39 Becker, Rolf: Educational Expansion and Persistent Inequalities of Education. Utilizing Subjective Expected Utility Theory to Explain Increasing Participation Rates in Upper Secondary School in the Federal Republic of Germany, in: European Sociological Review 19 (2003), No. 1, pp. 1–24, here p. 2.

40 Breen: Inequality (see footnote 38), p. 1494.

41 Barone/Ruggera: Equalization (see footnote 30).

42 Ibid., figure 1 on p. 12.

43 Schmidt: Retrenchment (see footnote 28), p. 174.

44 Ibid., pp. 168–170.

45 Barone/Ruggera’s data does not cover the period after the financial crisis of 2008, which hit Greece especially hard, causing the Greek economy to shrink by no less than a third and leading to drastic cuts in education spending of over 40 per cent between 2009 and 2015. See Dassiou, Xeni: Greece in Economic Crisis. The Case of Health and Education, in: Vierteljahrshefte zur Wirtschaftsforschung 84 (2015), No. 3, pp. 145–164. Such a perfect storm of grave economic depression, spiralling unemployment and severe retrenchment of public spending makes Greece an ideal-typical case study for future research on the development of educational inequalities.

46 Bukodi, Erzsébet/Goldthorpe, John H.: Decomposing ‘Social Origins’. The Effects of Parents’ Class, Status, and Education on the Educational Attainment of Their Children, in: European Sociological Review 29 (2013), No. 5, pp. 1024–1039.

47 Bukodi, Erzsébet/Erikson, Robert/Goldthorpe, John H.: The Effects of Social Origins and Cognitive Ability on Educational Attainment. Evidence from Britain and Sweden, in: Acta Sociologica 57 (2014), No. 4, pp. 293–310.

48 Barone/Ruggera: Equalization (see footnote 30), p. 16. Further research could clarify whether this disadvantage forms part of a persistent inequality of access to education and other resources between urban and rural areas. A study by Blank/Graham/Calvino into the local geographies of internet use argues that—at least in Britain—demographic and not geographic characteristics best predict access quality. See Blank, Grant/Graham, Mark/Calvino, Claudio: Local Geographies of Digital Inequality, in: Social Science Computer Review 36 (2018), No. 1, pp. 82–102.

49 Deer, Cécile: Different Paths, Similar Effects. Persistent Inequalities and their Sources in European Higher Education, in: Holsinger, Donald B./Jacob, W. James (eds.): Inequality in Education. Comparative and International Perspectives, Springer, New York 2008, pp. 324–347.

50 Simmons, Christina/Leigh, Lora: Making Marriage Modern. Women’s Sexuality from the Progressive Era to World War II, Oxford UP, Oxford 2009, pp. 138–177; Clark, David: Ideologies of Marriage and Family Life, in: Clark, David (ed.): Marriage, Domestic Life, and Social Change. Writings for Jacqueline Burgoyne 1944–88, Routledge, London/New York 1991, pp. 97–116.

51 Schwartz, Christine R./Mare, Robert D.: Trends in Educational Assortative Marriage from 1940 to 2003, in: Demography 42 (2005), No. 4, pp. 621–646. The trend was strong but not universal with socially conservative men in industrialised countries and most of the male population in threshold countries still marrying women with educational attainment lower than their own. See Blossfeld, Hans-Peter: Educational Assortative Marriage in Comparative Perspective, in: Annual Review of Sociology 35 (2009), pp. 513–530, here pp. 521f.

52 Schwartz, Christine R.: Earnings Inequality and the Changing Association between Spouses’ Earnings, in: American Journal of Sociology 115 (2010), No. 5, pp. 1524–1557; Smits, Jeroen/Park, Hyunjoon: Five Decades of Educational Assortative Mating in 10 East Asian Societies, in: Social Forces 88 (2009), No. 1, pp. 227–255.

53 Lyken-Segosebe, Dawn/Hinz, Serena E.: The Politics of Parental Involvement. How Opportunity Hoarding and Prying Shape Educational Opportunity, in: Peabody Journal of Education 90 (2015), No. 1, pp. 93–112. Tilly, Charles: Changing Forms of Inequality, in: Sociological Theory 21 (2003), No. 1, pp. 31–36, originally used the term to describe how cliques exclude non-members from the acquisition of resources. More recent studies have used the term to explore the dynamics of social inequality in a broader sense, especially in relation to education. See Rury, John L./Saatcioglu, Argun: Opportunity Hoarding, in: Stone, John et al. (eds.): The Wiley Blackwell Encyclopedia of Race, Ethnicity, and Nationalism, Emerald, Hoboken, NJ 2016, pp. 1–3.

54 For this general tendency see Lareau, Annette: Unequal Childhoods, California UP, Berkeley, CA et al. 2003.

55 Aktürk, Şener: Comparative Politics of Exclusion in Europe and the Americas. Religious, Sectarian, and Racial Boundary Making Since the Reformation, in: Comparative Politics 52 (2020), No. 4, pp. 695–719. As examples for European case studies see Rokem, Jonathan/Vaughan, Laura: Geographies of Ethnic Segregation in Stockholm. The Role of Mobility and Co-Presence in Shaping the ‘Diverse’ City, in: Urban Studies 56 (2019), No. 12, pp. 2426–2446; Lesger, Clé/Van Leeuwen, Marco: Residential Segregation from the Sixteenth to the Nineteenth Century. Evidence from the Netherlands, in: Journal of Interdisciplinary History 42 (2012), No. 3, pp. 333–369. For a methodologically innovative study with a neighbour-based approach to residential segregation in the US see Logan, Trevon D./Parman, John M.: The National Rise in Residential Segregation, in: The Journal of Economic History 77 (2017), No. 1, pp. 127–170.

56 Hamnett, Chris/Butler, Tim: Distance, Education and Inequality, in: Comparative Education 49 (2013), No. 3, pp. 317–330.

57 For the British case see Fitz, John: Schools, Markets and Choice Policies, Routledge, London 2003, pp. 111f.; for the US see Holme, Jennifer Jellison: Buying Homes, Buying Schools. School Choice and the Social Construction of School Quality, in: Harvard Educational Review 72 (2002), No. 2, pp. 177–206; Levin, Henry R.: An Economic Perspective on School Choice, in: Berends, Mark et al. (eds.): Handbook of Research on School Choice, Routledge, New York 2009, pp. 19–34.

58 Rury, John L./Saatcioglu, Argun: Suburban Advantage. Opportunity Hoarding and Secondary Attainment in the Postwar Metropolitan North, in: American Journal of Education 117 (2011), No. 3, pp. 307–342.

59 In his pioneering study dating from 1993, Jeffrey Mirel documented this dynamic in the case of Detroit. See Mirel, Jeffrey: The Rise and Fall of an Urban School System. Detroit 1907–81, Michigan UP, Ann Arbor, MI ^2^1999.

60 Rury/Saatcioglu: Advantage (see footnote 58), here p. 310.

61 See as an example for the accumulative effect of disadvantages on children’s intelligence Sampson, Robert J. et al.: Durable Effects of Concentrated Disadvantage on Verbal Ability Among African-American Children, in: Proceedings of the National Academy of Sciences 105 (2008), No. 3, pp. 845–852.

62 See Maloutas, Thomas/Ramos Lobato, Isabel: Education and Social Reproduction. Educational Mechanisms and Residential Segregation in Athens and Dortmund, in: Local Economy 30 (2015), No. 7, pp. 800–817.

63 See the case studies in Plank, David Nathan/Sykes, Gary (eds.): Choosing Choice. School Choice in International Perspective, Teachers College Press, New York/London 2003.

64 Holme: Homes (see footnote 57), p. 178.

65 Windle, Joel: The Rise of School Choice in Education Funding Reform. An Analysis of Two Policy Moments, in: Educational Policy 28 (2014), No. 2, pp. 306–324.

66 Rowe, Emma E./Lubienski, Christopher: Shopping for Schools or Shopping for Peers. Public Schools and Catchment Area Segregation, in: Journal of Education Policy 32 (2017), No. 3, pp. 340–356.

67 Bunar, Nihad: Choosing for Quality or Inequality. Current Perspectives on the Implementation of School Choice Policy in Sweden, in: Journal of Education Policy 25 (2010), No. 1, pp. 1–18.

68 Ramos Lobato, Isabel/Groos, Thomas: Choice As a Duty? The Abolition of Primary School Catchment Areas in North Rhine-Westphalia/Germany and its Impact on Parent Choice Strategies, in: Urban Studies 56 (2019), No. 15, pp. 3274–3291.

69 Ramos Lobato, Isabel/Bernelius, Venla/Kosunen, Sonja: Looking for the Ordinary? Parental Choice and Elite School Avoidance in Finland and Germany, in: Nordic Journal of Studies in Educational Policy 4 (2018), No. 3, pp. 156–167.

70 Allen, Kim et al.: Becoming Employable Students and ‘Ideal’ Creative Workers. Exclusion and Inequality in Higher Education Work Placements, in: British Journal of Sociology of Education 34 (2013), No. 3, pp. 431–452; Bathmaker, Ann-Marie et al.: Higher Education, Social Class and the Mobilisation of Capitals. Recognising and Playing the Game, in: British Journal of Sociology of Education 34 (2013), No. 5–6, pp. 723–743.

71 Van de Werfhorst, Herman G./Andersen, Robert: Social Background, Credential Inflation and Educational Strategies, in: Acta Sociologica 48 (2005), No. 4, pp. 321–340; Reay, Diane: ‘We never get a fair chance’. Working-Class Experiences of Education in the Twenty-First Century, in: Atkinson, Will/Roberts, Steven/Savage, Mike: Class Inequality in Austerity Britain. Power, Difference and Suffering, Palgrave Macmillan, London 2013, pp. 33–50.

72 Wilkinson, Richard/Pickett, Kate: The Spirit Level. Why Equality is Better for Everyone, Penguin, London 2009; eid.: The Inner Level. How More Equal Societies Reduce Stress, Restore Sanity and Improve Everyone’s Wellbeing, Penguin, London 2018.

73 See as an example, Snowdon, Christopher: The Spirit Level Delusion. Fact-Checking the Left’s New Theory of Everything, Democracy Institute, Ripon/London 2010.

74 See Chamberlain, Alyssa W./Hipp, John R.: It’s All Relative. Concentrated Disadvantage Within and Across Neighborhoods and Communities, and the Consequences for Neighborhood Crime, in: Journal of Criminal Justice 43 (2015), No. 6, pp. 431–443.

75 To name two influential publications, Kelly, Morgan: Inequality and Crime, in: Review of Economics and Statistics 82 (2000), No. 4, pp. 530–539, argued based on data from US-American urban counties that rising inequality does not affect property crimes. At the same time, it strongly correlates with an increasing rate of violent crimes. He theorised a reversed effect for poverty, with a substantial effect on property crimes but only a minor one on violent crimes. However, a meta-analysis of 17 papers in the field by Rufrancos, Hector et al.: Income Inequality and Crime. A Review and Explanation of the Time-Series Evidence, in: Sociology and Criminology 1 (2013), No. 1, pp. 1–9, concluded that rising income inequality goes along with an increase in both property crimes and violent crimes, with the former link being significantly more robust than the latter.

76 Cheong, Tsun Se/Wu, Yanrui: Crime Rates and Inequality. A Study of Crime in Contemporary China, in: Journal of the Asia Pacific Economy 20 (2015), No. 2, pp. 202–223.

77 For an influential example see Fajnzylber, Pablo/Lederman, Daniel/Loayza, Norman: Inequality and Violent Crime, in: The Journal of Law and Economics 45 (2002), No. 1, pp. 1–39.

78 See Frohmann, Lisa/Mertz, Elizabeth: Legal Reform and Social Construction. Violence, Gender, and the Law, in: Law & Social Inquiry 19 (1994), No. 4, pp. 829–851.

79 Van Eijk, Gwen: Socioeconomic Marginality in Sentencing. The Built-in Bias in Risk Assessment Tools and the Reproduction of Social Inequality, in: Punishment & Society 19 (2017), No. 4, pp. 463–481.

80 For an overview of these methodological controversies see Schneider, Simone M.: Income Inequality and Subjective Wellbeing. Trends, Challenges, and Research Directions, in: Journal of Happiness Studies 17 (2016), No. 4, pp. 1719–1739.

81 Cheung, Felix: Income Redistribution Predicts Greater Life Satisfaction Across Individual, National, and Cultural Characteristics, in: Journal of Personality and Social Psychology 115 (2018), No. 5, p. 867–882, argues on the basis of German as well as cross-country comparative data that lowering social inequality by 10 per cent increases overall happiness as much as a 37 per cent increase in overall prosperity. Similarly, Amendola, Adalgiso/Dell’Anno, Roberto/Parisi, Lavinia: Happiness and Inequality in European Countries. Is it a Matter of Peer Group Comparisons?, in: Economia Politica 36 (2019), No. 2, pp. 473–508, point out that people who live in countries with strong welfare states experience less adverse effects of inequality on their wellbeing. Contrary to these pro-redistribution arguments, Cullis, John/Hudson, John/Jones, Philip: A Different Rationale for Redistribution. Pursuit of Happiness in the European Union, in: Journal of Happiness Studies 12 (2011), No. 2, pp. 323–341, claim the impact of income redistribution on average national happiness in EU countries to be marginal.

82 Psychologists have a different approach to happiness than social scientists who are working in this field. While psychologists also acknowledge the impact of objective life circumstances on individual wellbeing, they stress that emotional happiness is more independent from material influences like income than a cognitive assessment of their life-satisfaction. See Decancq, Koen/Fleurbaey, Marc/Schokkaert, Erik: Chapter 2 Inequality, Income, and Well-Being, in: Atkinson, Anthony B./Bourguignon, François (eds.): Handbook of Income Distribution. Vol. 2A, Elsevier, London et al. 2015, pp. 67–140, here p. 85.

83 Frey, Bruno S./Stutzer, Alois: What Can Economists Learn from Happiness Research?, in: Journal of Economic Literature 40 (2002), No. 2, pp. 402–435.

84 See Delhey, Jan/Kohler, Ulrich: Is Happiness Inequality Immune to Income Inequality? New Evidence through Instrument-Effect-Corrected Standard Deviations, in: Social Science Research 40 (2011), No. 3, pp. 742–756.

85 For the data see Stevenson, Betsey/Wolfers, Justin: Happiness Inequality in the United States, in: The Journal of Legal Studies 37 (2008), No. S2, pp. S33–S79, at p. S40.

86 Gottschalk, Peter/Smeeding, Timothy M.: Cross-National Comparisons of Earnings and Income Inequality, in: Journal of Economic Literature 35 (1997), No. 2, pp. 633–687.

87 Alesina, Alberto/Di Tella, Rafael/MacCulloch, Robert: Inequality and Happiness. Are Europeans and Americans Different?, in: Journal of Public Economics 88 (2004), No. 9–10, pp. 2009–2042.

88 Alderson, Arthur/Katz-Gerro, Tally: Compared to Whom? Inequality, Social Comparison, and Happiness in the United States, in: Social Forces 95 (2016), No. 1, pp. 25–54.

89 Brown-Iannuzzi, Jazmin L. et al.: Subjective Status Shapes Political Preferences, in: Psychological Science 26 (2015), No. 1, pp. 15–26.

90 Amendola/Dell’Anno/Parisi: Happiness (see foonote 81).

91 For a more extensive argument in favour of such an approach see Pleinen, Jenny/Raphael, Lutz: Zeithistoriker in den Archiven der Sozialwissenschaften. Erkenntnispotenziale und Relevanzgewinne für die Disziplin, in: Vierteljahrshefte für Zeitgeschichte 62 (2014), No. 2, pp. 173–196.

92 During the early 1990s, Fukuyama became a world-famous public intellectual for the neoconservative movement with his end-of-history thesis. According to Fukuyama at that time, with the collapse of the Soviet Union world history had inevitably entered a period in which Western democracy and liberal world order would become universal, at least in the long run. For this argument see Fukuyama, Francis: The End of History?, in: The National Interest 16 (1989), pp. 3–18, as well as his follow-up book Fukuyama, Francis: The End of History and the Last Man, Penguin, Harmondsworth 1992. For the role of this discourse in the development of US neoconservative foreign policy see Schmidt, Brian C./Williams, Michael C.: The Bush Doctrine and the Iraq War. Neoconservatives versus Realists, in: Security Studies 17 (2008), No. 2, pp. 191–220.

93 Fukuyama, Francis: Identity. The Demand for Dignity and the Politics of Resentment, Farrar Straus & Giroux, New York 2018, pp. 6–11.

94 For an integrated approach to a history of emotions and its methodological implications see Lang, Johannes: New Histories of Emotion, in: History & Theory 57 (2018), No. 1, pp. 104–120.

95 Schumacher, Gijs/Van Kersbergen, Kees: Do Mainstream Parties Adapt to the Welfare Chauvinism of Populist Parties?, in: Party Politics 22 (2016), No. 3, pp. 300–312.

96 Gonthier, Fréderic: Parallel Publics? Support for Income Redistribution in Times of Economic Crisis, in: European Journal of Political Research 56 (2017), No. 1, pp. 92–114.

97 Oesch, Daniel: Explaining Workers’ Support for Right-Wing Populist Parties in Western Europe. Evidence from Austria, Belgium, France, Norway, and Switzerland, in: International Political Science Review 29 (2008), No. 3, pp. 349–373.

98 Oesch also points out that workers lost traditional channels of representation and influence as a result of social disorganisation and therefore experience political alienation. However, he does not explain how political alienation links to cultural anxieties. See ibid., p. 350.

99 See Burgmann, Verity: From Syndicalism to Seattle. Class and the Politics of Identity, in: International Labor and Working-Class History 67 (2005), pp. 1–21; Savage, Mike: Review Essay. A New Class Paradigm?, in: British Journal of Sociology of Education 24 (2003), No. 4, pp. 535–541.

100 See Manow, Philip/Schwander, Hanna/Palier, Bruno: Conclusions. Electoral Dynamics in Times of Changing Welfare Capitalism, in: eid. (eds.): Welfare Democracies and Party Politics. Explaining Electoral Dynamics in Times of Changing Welfare Capitalism, Oxford UP, Oxford 2018, pp. 298–314.

101 Coenders, Marcel/Scheepers, Peer: Changes in Resistance to the Social Integration of Foreigners in Germany 1980–2000. Individual and Contextual Determinants, in: Journal of Ethnic and Migration Studies 34 (2008), No. 1, pp. 1–26.

102 Van Der Waal, Jeroen/De Koster, Willem/Van Oorschot, Wim: Three Worlds of Welfare Chauvinism? How Welfare Regimes Affect Support for Distributing Welfare to Immigrants in Europe, in: Journal of Comparative Policy Analysis. Research and Practice 15 (2013), No. 2, pp. 164–181.

103 Abreu, Maria/Öner, Özge: Disentangling the Brexit Vote. The Role of Economic, Social and Cultural Contexts in Explaining the UK’s EU Referendum Vote, in: Environment and Planning A. Economy and Space 52 (2020), No. 7, pp. 1434–1456.

104 Bakir, Vian: Psychological Operations in Digital Political Campaigns. Assessing Cambridge Analytica’s Psychographic Profiling and Targeting, in: Frontiers in Communication 67 (2020), No. 5, pp. 1–16.

105 For the effects of globalization on regional inequalities during the 1990s and 2000s see Ezcurra, Roberto/Del Villar, Alba: Globalization and Spatial Inequality. Does Economic Integration Affect Regional Disparities?, in: The Annals of Regional Science (2021), No. 2, pp. 1–24.

106 Tomlinson, Jim: Managing the Economy, Managing the People. Narratives of Economic Life in Britain from Beveridge to Brexit, Oxford UP, Oxford et al. 2017, pp. 107–109.

107 Abreu/Öner: Brexit (see footnote 103), p. 1445.

108 This strategy was aided by a news coverage that depicted Turkish migrants in a very negative light. See Ker-Lindsay, James: Turkey’s EU Accession as a Factor in the 2016 Brexit Referendum, in: Turkish Studies 19 (2018), No. 1, pp. 1–22, here p. 11.

109 For an overview of this debate see Carnes, Nicholas/Lupu, Noam: The White Working Class and the 2016 Election, in: Perspectives on Politics 19 (2021), No. 1, pp. 55–72.

110 Zake, Ieva: Nixon vs. the GOP. Republican Ethnic Politics. 1968–1972, in: Polish American Studies 67 (2010), No. 2, pp. 53–74.

111 Griffin, Robert/Teixeira, Ruy: The Story of Trump’s Appeal. A Portrait of Trump Voters. A Research Report by the Democracy Fund Voter Study Group, June 2017, URL: <https://www.voterstudygroup.org/publication/story-of-trumps-appeal> [accessed: 30.07.2021], p. 4.

112 Manza, Jeff/Crowley, Ned: Working Class Hero? Interrogating the Social Bases of the Rise of Donald Trump, in: The Forum 15 (2017), No. 1, pp. 3–28, here p. 23. The article compares the median income of Trump voters to the national average (73,000 versus 56,000$). The national median (without Puerto Rico) for the year 2016 was 59,039$ (for the data see Semega, Jessica L./Fontenot, Kayla R./Kollar, Melissa A.: Income and Poverty in the United States: 2016, United States Census Bureau, Washington, DC 2017, URL: <https://www.census.gov/library/publications/2017/demo/p60-259.html> [accessed: 03.08.2021]).

113 Smith, David Norman/Hanley, Eric: The Anger Games. Who Voted for Donald Trump in the 2016 Election, and Why?, in: Critical Sociology 44 (2018), No. 2, pp. 195–212.

114 See McQuarrie, Michael: The Revolt of the Rust Belt. Place and Politics in the Age of Anger, in: The British Journal of Sociology 68 (2017), No. S1, pp. S120–S152.

115 Van Der Waal/De Koster/Van Oorschot: Worlds (see footnote 102).

116 For the original theory see Korpi, Walter: The Democratic Class Struggle, Routledge, London 1983.

117 Edlund, Jonas/Lindh, Arvid: The Democratic Class Struggle Revisited. The Welfare State, Social Cohesion and Political Conflict, in: Acta Sociologica 58 (2015), No. 4, pp. 311–328.

118 Clark, Gregory: The Son also Rises. Surnames and the History of Social Mobility, Princeton UP, Princeton, NJ/Oxford 2014, p. 12.

119 Mare, Robert D.: A Multigenerational View of Inequality, in: Demography 48 (2011), No. 1, pp. 1–23.

120 Chan, Tak Wing/Boliver, Vikki: The Grandparents’ Effect in Social Mobility. Evidence from British Birth Cohort Studies, in: American Sociological Review 78 (2013), No. 4, pp. 662–678.

121 Levy, Frank/Michel, Richard C.: The Economic Future of American Families. Income and Wealth Trends, Urban Institute Press, Washington, DC 1991, p. 59.

122 It is, however, disputed whether the grandparents’ effect consists predominantly in the transmission of economic, or of cultural and social, capital, as Stine Møllegaard and Mads Meier Jæger have argued for Scandinavia; see Møllegaard, Stine/Jæger, Mads Meier: The Effect of Grandparents’ Economic, Cultural, and Social Capital on Grandchildren’s Educational Success, in: Research in Social Stratification and Mobility 42 (2015), pp. 11–19.

123 Clark: Son (see footnote 118), p. 73.

124 Maas, Ineke: Review of the Book *The Son also Rises. Surnames and the History of Social Mobility* by Gregory Clark, in: Economic History Review 68 (2015), No. 4, pp. 1481f.

125 The only exception Clark allows are policies that influence the marriage rate between social groups with different levels of abilities. See Clark: Son (see footnote 118), p. 14.

126 Herrnstein, Richard J./Murray, Charles: The Bell Curve. Intelligence and Class Structure in American Life, Free Press, New York et al. 1994. Considering its political importance, the Bell Curve Debate has received relatively little attention from historians.

127 At the heart of this reform was the ”Personal Responsibility and Work Opportunity Act” of 1996, which ended entitlement to welfare payments, implemented *workfare* principles and enabled states to privatise social services. See Graebner, William: The End of Liberalism. Narrating Welfare’s Decline, from the Moynihan Report (1965) to the Personal Responsibility and Work Opportunity Act (1996), in: Journal of Policy History 14 (2002), No. 2, pp. 170–190.

